# Group Metacognitive Therapy for Generalized Anxiety Disorder: A Pilot Feasibility Trial

**DOI:** 10.3389/fpsyg.2019.00290

**Published:** 2019-02-14

**Authors:** Svein Haseth, Stian Solem, Grethe Baardsen Sørø, Eirin Bjørnstad, Torun Grøtte, Peter Fisher

**Affiliations:** ^1^Nidaros DPS, St. Olavs Hospital, Trondheim, Norway; ^2^Department of Psychology, Norwegian University of Science and Technology, Trondheim, Norway; ^3^Department of Research and Development, St. Olavs Hospital, Trondheim, Norway; ^4^Institute of Psychology, Health and Society, University of Liverpool, Liverpool, United Kingdom

**Keywords:** metacognitive therapy, generalized anxiety disorder, GAD, outcome, metacognition, group therapy

## Abstract

**Background:** Individual metacognitive therapy (MCT) for generalized anxiety disorder (GAD) is well established, but only one study has investigated the effectiveness of Group MCT (g-MCT) for GAD. The aim of the current study was therefore to evaluate the feasibility and effectiveness of g-MCT for GAD within a community mental health setting whilst addressing limitations evident in the previous study.

**Methods:** The study used an open trial design, and 23 consecutively referred adults with GAD completed 10 sessions (90 min) of g-MCT, delivered by two therapists trained in MCT. Diagnoses were assessed by trained raters using the Anxiety Disorder Interview Schedule-IV. All patients but one had previous psychosocial treatment, and 17 (73.9%) had at least one comorbid axis-I disorder. Self-reported symptoms were assessed using the Penn State Worry Questionnaire, the Generalized Anxiety Disorder-7, and the Patient Health Questionnaire-9 at pre- and post-treatment as well as 3-month follow-up. Feasibility was assessed using rates of patients who declined group treatment in favor of individual treatment, patients not able to attend due to pre-scheduled dates for sessions, and drop-out rate.

**Results:** Of 32 eligible participants, six patients (19%) declined g-MCT in favor of individual MCT, and three (9%) were unable to attend due to scheduling conflicts. No patients dropped out during treatment, but two patients did not complete the self-report questionnaires at 3-month follow-up. g-MCT was associated with significant reductions in worry, anxiety, depression, metacognitive beliefs, and maladaptive coping. According to the standardized Jacobson criteria for recovery, 65.3% were recovered at post-treatment, whereas 30.4% were improved and 4.3% showed no change. At 3-month follow-up, the recovery rate increased to 78.3%. Moreover, recovery rates were comparable for patients with- and without comorbidity. Number of therapist hours per patient was 6.5 and the treatment has now been implemented as a standard treatment option at the clinic.

**Conclusion:** g-MCT for GAD is an acceptable treatment which may offer a cost-effective alternative approach to individual MCT. Recovery rates and effect sizes suggested that g-MCT could be just as efficient as individual MCT and cognitive behavioral therapy.

## Introduction

Generalized anxiety disorder (GAD) is a common disorder associated with a chronic course and significantly reduced quality of life (Spitzer et al., 2006; American Psychiatric Association, 2013). It is characterized by excessive and uncontrollable worry related to multiple events or activities, with a duration of six months or more (American Psychiatric Association, 2013). Associated symptoms include restlessness, fatigue, difficulties concentrating, irritability, muscle tension, and sleep difficulties (American Psychiatric Association, 2013).

Cognitive behavioral therapy (CBT) is currently an evidence-based treatment for GAD (Hoyer et al., 2011). Meta-analyses show that CBT leads to a reduction in anxiety symptoms more so than treatment as usual or a waiting list (Mitte, 2005; Hunot et al., 2007; Covin et al., 2008). However, based on the criteria for clinically significant change (Jacobson and Truax, 1991), only 50–60% of patients with GAD recover at 6-month follow-up after CBT (Fisher and Durham, 1999). Thus, since a considerable proportion of GAD patients do not recover following CBT, more effective interventions are required.

Metacognitive Therapy (MCT) for GAD is an alternative treatment to CBT. MCT focuses on changing thought processes rather than thought content (e.g., Wells, 1995). MCT is derived from the self-regulatory executive function (S-REF) model (Wells and Matthews, 1994, 1996). Maintenance of psychological problems is linked to the activation of the cognitive-attentional syndrome (CAS) consisting of repetitive thinking (worry and rumination), threat monitoring, and maladaptive coping behaviors. The CAS is a product of an individual’s metacognitive beliefs and knowledge. Central to the metacognitive model of GAD (Wells, 1995, 1997, 2009) is that individuals’ thoughts and beliefs about worry (i.e., metacognitive beliefs) contribute to the development and maintenance of the disorder. Worry is often triggered by negative intrusive thoughts in the form of “what if” questions, e.g., “What if I’m involved in an accident?”. Thereafter, the use of worry is related to the activation of positive metacognitive beliefs about the advantages or benefits of worrying (Wells, 2009). Examples of such positive beliefs are “Worrying makes me prepared, and focusing on threat keep me safe.”

Symptoms of GAD escalate when negative metacognitive beliefs about worry are activated. Two types of negative beliefs are important: negative beliefs about the uncontrollability of worry (e.g., “I have lost control over my thoughts”) and negative beliefs about the possible dangers of worry (“If I do not stop worrying, I will lose my mind”). The activation of negative metacognitive beliefs leads to worry about worry (also called “meta-worry” or “Type 2-worry”), which intensifies worry, anxiety, and other maladaptive coping strategies. The model proposes that individuals with GAD tend to use worry as a coping strategy to safeguard against perceived threats and dangers. Examples of other frequently used coping responses among GAD patients are thought suppression, threat monitoring, distraction, avoidance, and reassurance seeking. These coping strategies backfire and consolidate the belief that worry is uncontrollable.

The metacognitive model of GAD (Wells, 1995, 1997, 2009) proposes that both positive and negative metacognitive beliefs need to be modified to enable people to disengage from worrying in response to trigger thoughts. Furthermore, the model specifies that counterproductive coping strategies need to be modified if people are to successfully reduce worry.

So far, four studies have evaluated MCT for GAD delivered individually for outpatients. Wells and King (2006) conducted an open trial (*N* = 10), where a range of 3–12 weekly MCT sessions were delivered. There were significant improvements in symptoms of worry, anxiety, and depression at post-treatment [within-group *d*’s between 1.12 (health worry) and 2.78 (trait-anxiety)] and follow-up (within-group *d*’s between 1.10 and 2.58), and 87.5% of the patients met criteria for recovery on trait-anxiety (STAI-T) at post-treatment, and 75% were recovered at 6- and 12-month follow-up.

The second study was conducted by Wells et al. (2010) and was a randomized controlled trial (*N* = 20, 10 in each condition) where MCT was compared with applied relaxation (AR) in patients with GAD. Treatment sessions lasted 45–60 min and were held once per week for 8–12 weeks. MCT was significantly more effective in reducing GAD symptoms than AR. Following criteria (Fisher and Durham, 1999) for clinically significant change (PSWQ; cut-off ≤47, reliable change index: 7), the recovery rate was 80% in the MCT group at post-treatment, compared with 10% in the AR group. At 6-month follow-up, the recovery rate was 70% in the MCT group and 10% in the AR group, while the figure was 80 and 10%, respectively, at 12-month follow-up. High recovery rates combined with a large within-group effect size (*d* = 3.41) indicated that MCT was an effective treatment for GAD.

van der Heiden et al. (2012) investigated the effectiveness of MCT and intolerance of uncertainty therapy (IUT). Each treatment consisted of a maximum of 14 weekly sessions of 45 min. Both MCT and IUT were associated with significant reductions in symptoms of GAD at post-treatment and 6-month follow-up, but MCT was found to be significantly superior to IUT. The within-group effect sizes for worry (PSWQ) in the MCT group were high at both post-treatment (*d* = 1.67) and follow-up (*d* = 1.66), and the between-group effect sizes were 0.96 at post-treatment and 0.78 at follow-up. In the MCT intention-to-treat group, 60% met criteria for recovery on PSWQ (cut-off ≤53, reliable change index: 7) at end of treatment and 62% at follow-up. The corresponding recovery rates for the IUT group were 37% and 47%, respectively.

Nordahl et al. (2018) compared the efficacy of MCT and CBT for GAD. Both CBT and MCT produced significant reductions in worry (PSWQ) in comparison to the wait list group. However, MCT was found to be more effective than CBT. In the MCT condition 65% were classified as recovered post-treatment in comparison to 38% in the CBT condition, and the difference was maintained at 2-year follow-up.

In summary, previous research indicates that individual outpatient MCT for GAD is well established. According to, the (National Institute for Health and Clinical Excellence [NICE], 2011) guidelines, MCT is a recommended treatment for GAD. However, group MCT (g-MCT) for GAD has only been examined in one open trial (van der Heiden et al., 2013). This study used large groups (10–14 patients) which may limit participation of some group members and not allow therapy to be implemented with sufficient specificity to address individual needs. In addition, two out of the four therapists had not received training in MCT thereby potentially limiting treatment adherence and competency. The sample consisted of 33 outpatients, treatment sessions lasted 90 min and were held weekly for 12–14 weeks. There were significant reductions in worry, anxiety, and negative metacognitive beliefs. In the intention-to-treat sample, the between group effect sizes at post-treatment and 6-month follow-up were 1.24 and 1.29, respectively. In terms of recovery, 55% of participants met criteria for clinically significant criteria at post-treatment recovery rate at post-treatment (cut-off: ≤53, reliable change index: 7).

Treatment in a group can be an attractive alternative to individual treatment for several reasons. A similar effect as individual treatment will result in group treatment being more cost-effective by cutting down on long waiting lists leading to more effective use of the therapists’ time. One assumption is that MCT will be well-suited to a group format because it is based on a transdiagnostic model. A recent study supported the use of g-MCT for a transdiagnostic sample (Capobianco et al., 2018). The study found that g-MCT was more effective than Mindfulness Based Stress Reduction in treating symptoms of anxiety and depression. Furthermore, patients with GAD may worry about different events, activities, life events and will frequently have different comorbid disorders, but MCT focuses on changing the attitudes and beliefs one has around thought processes (i.e., worrying and rumination) and is less concerned with the actual idiosyncratic thought content of each patient. Patients can help each other identify shared maladaptive metacognitive beliefs and coping strategies whilst their worry content differ.

Despite the appealing aspect of group treatment, a comparison of effect sizes, recovery-, and attrition rates with previous studies of individual MCT indicates that g-MCT may be less effective. Furthermore, the dropout rate was higher in g-MCT (27%) than in individual treatment studies (van der Heiden et al., 2012: 18%; Wells and King, 2006 and Wells et al., 2010: 0%). In addition to the limitations of the van der Heiden et al. (2013) study, the authors also suggested several possible reasons for the differences from individual MCT. First, the large group size (10–14 patients per group) may have reduced the acceptability of the treatment modality and contributed to the high drop-out rate. Second, there may have been less time to identify and challenge each patient’s idiosyncratic metacognitive beliefs, given the group size. Third, therapist factors may have comprised the effectiveness of the intervention as only two out of four therapists were trained in MCT, and there was no supervision in delivering g-MCT.

In summary, even though van der Heiden et al’s. (2013) results indicated that g-MCT was effective in reducing GAD symptoms, many questions remain regarding the feasibility of g-MCT, such as recruitment, group size, and retention. Consequently, the primary aim of the current study was to benchmark and evaluate the feasibility of g-MCT for adult patients with GAD. Moreover, to explore whether smaller groups would be more feasible and effective, as only 4–6 patients were included in each group. The study was conducted at a Norwegian psychiatric outpatient clinic without a control group. The secondary aim of the study was to evaluate the effectiveness of g-MCT, with the hypothesis being that g-MCT will be associated with significant reductions in symptoms of GAD and depression, as well as reductions in positive- and negative metacognitions, maladaptive coping strategies, and avoidance.

## Materials and Methods

### Participants

The sample consisted of 23 participants, of which 22 were women (95.7%). The average age was 29.70 years (*SD* = 9.21). Further demographic characteristics are shown in Table 1. The four patients using antidepressants reported to use either Zoloft or Cipralex. Three of these four had been on a stable dose for years, while the fourth started medication 4 months before treatment. No changes were made to medication during treatment. In addition, two patients used medicine for sleep related problems.

**Table 1:** Demographic and diagnostic characteristics of the sample (*N* = 23).

	*n*	%
Female	22	95.7
Single	7	30.4
Married/cohabitant	16	69.6
Full time employed	11	47.8
Student	8	34.8
Welfare benefits	4	17.4
Current use of antidepressants	4	17.4
Previous psychiatric outpatient treatment	22	95.7
**Comorbidity**		
Obsessive-compulsive disorder	6	26.1
Depression	4	17.4
Panic disorder	4	17.4
Social anxiety disorder	2	8.7
Specific phobia	1	4.3
Health anxiety	1	4.3
ADHD	2	8.7


*Patients diagnosed with ADHD were already diagnosed with ADHD as described in their referral.*

Diagnosis was established using the Anxiety Disorder Interview Schedule (ADIS-IV, Brown et al., 1994). To be included in the present study, GAD had to be the primary diagnosis. None of the participants had known serious somatic illnesses, psychosis, post-traumatic stress disorder, known cluster A- or B personality disorders, were suicidal, or suffered from drug addiction. Seventeen (73.9%) participants had comorbid disorders. Fourteen had one comorbid disorder (OCD = 4, depression = 2, panic disorder = 3, social anxiety disorder = 1, specific phobia = 1, health anxiety = 1, ADHD = 2). Three patients had two comorbid diagnoses (one with panic disorder and depression, one with OCD and depression, and one with OCD and social phobia).

### Procedure

The clinic has a population catchment of approximately 130,000 people. Patients were referred to the clinical service from their GP, student health services, and mental health clinics. The first group started in September 2016 and the last group started in October 2017. Patients included in the study were consecutive referrals.

Pre-treatment assessment consisted of the ADIS-IV (Brown et al., 1994) and completion of self-report questionnaires. The ADIS-IV was conducted by independent investigators (clinical psychologists not involved with the treatment) trained in diagnostic interviewing. Patients received no treatment whilst waiting for treatment to start. The wait time period was 3–4 months.

Five groups were held, each with 4–6 patients. The groups were held at Nidaros DPS, St. Olavs Hospital. Patients were offered 10 weekly group sessions, each with a duration of 90 min. All self-report questionnaires were completed at pre-treatment, post-treatment, and at 3-month follow-up. The first groups completed questionnaires on pen and paper at the clinic, while the more recent groups completed questionnaires online. In addition, the Generalized Anxiety Disorder Scale-Revised (GADS-R; Wells, 2009) was distributed before the beginning of each treatment session. All subjects gave written informed consent in accordance with the Declaration of Helsinki. The study was approved by the Regional Committees for Medical and Health Research Ethics in Norway (REK; 2013/2155, Helse Midt, https://helseforskning.etikkom.no/) and conducted without external funding.

### Therapists

All groups were led by two therapists; a psychiatric nurse and a clinical psychologist. Both had completed training in MCT and were registered level 1 and level 2 therapists respectfully. Video supervision was conducted with a master clinician in MCT. Furthermore, several groups had been conducted for training purposes before the open trial was initiated.

### Treatment

The g-MCT had a specific structure and followed the treatment manual for GAD (Wells, 2009). Sessions one and two focused on creating a group case formulation. Participants were helped to create their own personal case formulation. Participants were socialized to the metacognitive model and introduced to the concept of detached mindfulness (detached mindfulness; Wells, 2009). Sessions three and four focused on challenging metacognitive beliefs regarding uncontrollability of worry and the belief that they would lose control if they worried too much. In order to clarify conflicting and dysfunctional metacognitions, the group was divided into two smaller groups and they constructed arguments for worry being controllable or not, and if they could lose control or not. The participants then discussed and challenged each other’s beliefs, with help from the therapists.

In sessions five and six the primary aim of MCT was to reduce negative beliefs about the dangers of worry. Both verbal and behavioral strategies were used to challenge metacognitions. Examples of verbal strategies were questioning the evidence of metacognitive beliefs and searching for counterclaims (as with beliefs about uncontrollability in earlier sessions). Thereafter, in session 7 and 8, positive beliefs about worry were challenged and modified.

The last phase of therapy (session 9 and 10) focused on relapse prevention. The group members made a summary of their case formulation (therapy blueprint) and a summary (“old and new plan”) of how they used to respond to negative thoughts in the past and contrasted this with their new adaptive responses to worrying thoughts.

### Measures

The Penn State Worry Questionnaire (PSWQ; Meyer et al., 1990) is a 16-item self-report questionnaire measuring the severity of worry, both in terms of frequency, intensity and uncontrollability. Each item is rated from 1 (“*not at all typical of me*”) to 5 (“*very typical of me*”). The total score ranges from 16 to 80, where a higher score indicates higher levels of pathological worry. It has excellent internal consistency (Cronbach α = 0.93) and good psychometric properties (Meyer et al., 1990). Cronbach’s alpha in the current study was 0.97.

Generalized Anxiety Disorder-7 (GAD-7; Spitzer et al., 2006) is a self-report questionnaire with seven items assessing symptoms of GAD. Patients answer how much during the last two weeks they have been bothered by each symptom. The answer options range from 0 (“*not at all*”) to 3 (“*almost every day*”), resulting in a total score between 0 and 21. A clinical cut-off point of 10 has been suggested. GAD-7 has been shown to have excellent internal consistency (Cronbach α = 0.92) and good test-retest reliability (*r* = 0.83). It has also demonstrated good criterion, construct, factorial, and procedural validity (Spitzer et al., 2006). Cronbach’s alpha in the current study was 0.89.

The Patient Health Questionnaire-9 (PHQ-9; Kroenke et al., 2001) is a self-report questionnaire designed to measure symptoms of depression using nine items corresponding to the nine criteria for depression. The patient answers how troublesome each problem has been during the past two weeks, where each question is scored on a scale of 0 (“*not at all*”) to 3 (“*almost every day*”). The total score range from 0 to 27, of where a cut point of 10 identifies major depression with good sensitivity and specificity (Kroenke et al., 2001). The PHQ-9 has demonstrated excellent internal reliability (Cronbach α = 0.86) and test-retest reliability, as well as good construct and convergent validity (Kroenke et al., 2001). Cronbach’s alpha in the current study was 0.90.

Generalized Anxiety Disorder Scale-Revised (Wells, 2009) is a self-report inventory based on the metacognitive model of GAD. The first items cover GAD symptoms, time spent worrying, as well as how often a range of coping and avoidance behavior have been done the last week. These items are scored on a scale from 0 to 8. In addition, the GADS-R assesses negative and positive metacognitive beliefs related to worry (Wells, 2009), each measured on a scale from 0 (“*I do not believe this at all*”) to 100 (“*I’m completely convinced this is true*”). Cronbach’s alpha for the coping items was 0.94, 0.79 for avoidance items, and 0.94 for the metacognitive belief items (0.94 for negative beliefs and 0.93 for positive).

### Data Analysis

**FIGURE 1:**
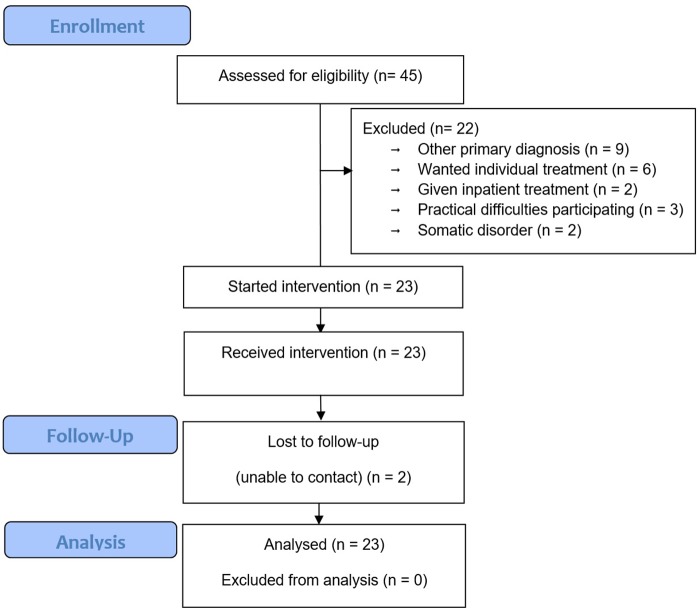
Flow chart.

The feasibility of g-MCT was operationalized and visualized through the participant flow chart (Figure 1), of where recruitment and retention rates are important feasibility outcomes. The results are contrasted with the g-MCT study of van der Heiden et al. (2013).

A repeated measures ANOVA was used to investigate changes in worry and symptoms of anxiety and depression. The same test was used to measure changes in metacognitions, coping strategies, and avoidance. There was no significant skewness or kurtosis on pre-treatment measures. Mauchly’s test of sphericity was not significant for all analyses using repeated measures ANOVA, except for PHQ-9, negative beliefs, and positive beliefs.

Effect sizes (Cohen, 1992) were calculated with Morris and Deshon’s equation no. 8, which controls the correlation between pre- and post-treatment values of the dependent variable. Following Jacobson and Truax (1991) and Fisher (2006), recovery (clinically significant change on the PSWQ) was calculated with the following criteria: cut-off = 47, reliable change index = 7. The study uses a cut-off point and a reliable change index that has been applied to a large group of GAD patients and use the standardized criteria as described in Fisher (2006). These criteria have been used in all other MCT studies for GAD except for the van der Heiden et al. (2013) study. Using the standardized criteria allows benchmarking of the results and allows a reasonable comparison between individual and group MCT. Along with effect sizes, recovery rates were used to compare the treatment effectiveness of the current study with previous studies of both individual and group based MCT for GAD.

Two patients did not complete questionnaires at follow-up. These values were replaced using last observation carried forward (one classified as improved and one as a treatment non-responder). There were no other missing values at pre-treatment, post-treatment, or follow-up. Missing values for session-to-session data were not replaced.

Lastly, the potential influence of comorbid disorders on treatment outcome was investigated using independent *t*-tests. The PSWQ, GAD-7, and PHQ-9 scores of patients with and without comorbid disorders were compared at pre-treatment, post-treatment, and 3-month follow-up.

## Results

### Feasibility

As shown in the participant flow chart (Figure 1), 45 patients were referred to and assessed for inclusion in the current study. Twenty-three patients were entered into the study and 22 patients were excluded. The most common reason for exclusion was that GAD was not the primary diagnosis (*n* = 9). Furthermore, two patients were excluded due to serious somatic disorder, and another two patients were given inpatient treatment instead of outpatient treatment because of their symptom severity and low level of functioning. Six patients preferred individual treatment instead of group treatment, and three patients could not participate in g-MCT due to practical difficulties. Therefore approximately 75% of suitable patients were included in the study. More specifically, 28.1% i.e., 9 of the 32 offered g-MCT declined.

Patients attended a mean of 8.9 (*SD* = 1.3) sessions. More specifically: one patient attended five sessions (due to scheduling conflicts), two received seven sessions, four received eight sessions, seven received nine sessions, and nine patients attended all ten sessions. Number of sessions were not significantly correlated with symptoms at post-treatment (*r* = 0.32 and *p* = 0.13) or follow-up (*r* = 0.35 and *p* = 0.10). Patients were asked to give their feedback on treatment acceptability in the tenth and final treatment session. For each group, all patients reported that they would have preferred group treatment rather than individual treatment because they were able to meet other patients which enabled them to learn from each other, and that the group setting reduced stigma related problems.

After completion of the open trial, the two therapists reported that delivering treatment in a group format was clinically appropriate and that the small group format need not prevent any patients from fully participating in the therapy. Furthermore, the clinicians plan to continue to use g-MCT in their routine clinical practice as it is cost-effective and reduces the length of time patients have to wait for treatment.

No patients dropped out during treatment, but two patients did not complete the self-report questionnaires at 3-month follow-up.

### Treatment Effect

Table 2 shows the mean and standard deviations for pre- and post-treatment scores and 3-month follow-up. A repeated measures ANOVA was conducted to investigate changes. Mauchley’s test was not significant on any of the analyses (except for PHQ-9, and negative- and positive metacognitions), and Wilks’ lambda was therefore used. The results show significant improvements and large effect sizes for all measures. Linear mixed model analysis was also attempted with these data. However, all slopes went in the same direction as the results were unambiguous. Furthermore, there were no significant fixed effects only a clear effect of time. Model fit did not significantly improve when including attendance rate and age into the model compared to a simple model.

**Table 2:** Repeated measures ANOVA testing change in symptoms and metacognitions.

	Pre	Post	F-U	*F*	Part Eta sq.	*d*	*d*
					
	*M(SD)*					*Post*	*Follow-up*
PSWQ	71.52 (5.97)	38.35 (14.02)	35.04 (13.71)	78.38^∗∗∗^	0.88	2.42	2.95
GAD-7	14.17 (3.97)	3.83 (3.38)	3.70 (2.77)	78.39^∗∗∗^	0.88	2.30	2.34
PHQ-9	13.87 (5.55)	4.70 (4.03)	4.91 (5.11)	32.15^∗∗∗^	0.75	1.76	1.38
GADS-R							
Negative	67.17 (21.70)	4.71 (12.62)	4.78 (12.50)	136.62^∗∗∗^	0.86	2.55	2.56
Positive	29.78 (25.87)	2.97 (6.19)	1.88 (4.06)	23.51^∗∗∗^	0.52	1.11	1.34
Coping	4.35 (1.21)	0.76 (0.90)	0.79 (0.84)	91.04^∗∗∗^	0.90	2.54	2.82
Avoidance	2.96 (1.31)	0.38 (0.67)	0.44 (0.68)	45.37^∗∗∗^	0.81	2.00	2.13


*Greenhouse–Geisser correction used for PHQ-9, and negative- and positive beliefs. Effect sizes (Cohen’s, 1992) were calculated using Morris and Deshon’s equation no. 8 controlling for correlation between pre- and post-treatment value for the variable in question. PSWQ, Penn State Worry Questionnaire; GAD-7, Generalized Anxiety Disorder-7; PHQ-9, Patient Health Questionnaire-9; GADS-R, Generalized Anxiety Disorder Scale-Revised.*

Changes in symptoms were significant from pre-treatment to post-treatment, and there were non-significant changes from post-treatment to follow-up for all three measures. In addition to tests of statistical significance, clinically significant change was investigated. Only one patient did not respond to treatment. A summary of recovery rates are displayed in Table 3.

**Table 3:** Recovery rates (percentages) at post-treatment and follow-up.

	Deterioration	No change	Improved	Recovered
**PSWQ**				
Post-treatment	0.0	4.3	30.4	65.3
Follow-up	0.0	4.3	17.4	78.3
**GAD-7**				
Post-treatment	0.0	4.3	8.7	87.0
Follow-up	0.0	0.0	21.7	78.3
**PHQ-9**				
Post-treatment	0.0	8.7	39.1	52.2
Follow-up	0.0	13.0	21.7	65.3


*PSWQ, Penn State Worry Questionnaire; GAD-7, Generalized Anxiety Disorder-7; PHQ-9, Patient Health Questionnaire-9. Cut-off values for GAD-7 and PHQ-9 was set at >10. Improved = at least 7-points improvement on PSWQ or below cut-off. Recovered = criterion for improved and scoring 47 or less on PSWQ. 91.3% of participants scored above cut-off on GAD-7 at pre-treatment, and 73.9% scored above cut-off on PHQ-9. The two patients that scored below cut-off on GAD-7 at pre-treatment were not classified as recovered (probably due to low pre-treatment values).*

Patients with comorbid disorders did not have significantly more symptoms than patients with no comorbidity at any of the three times of assessment. For PSWQ there was no significant difference at pre-treatment, *t*(21) = 0.96, *p* = 0.35, at post-treatment, *t*(21) = 1.82, *p* = 0.08, or follow-up, *t*(21) = 1.27, *p* = 0.22. Five of the six (83.3%) patients without comorbid disorders were recovered at follow-up compared to 76.5% for patients with comorbid disorders. For GAD-7 there was also no difference at pre-treatment, *t*(21) = 0.36, *p* = 0.73, at post-treatment, *t*(21) = 0.55, *p* = 0.73, or follow-up, *t*(21) = 0.71, *p* = 0.49. Same observation was made for PHQ-9 at pre-treatment, *t*(21) = 0.61, *p* = 0.55, at post-treatment, *t*(21) = 1.34, *p* = 0.19, and at follow-up, *t*(21) = 0.32, *p* = 0.76.

### Metacognitive Changes From Session to Session

Generalized Anxiety Disorder Scale-Revised was completed by patients before every session to measure changes in symptoms, worry, metacognitions, coping strategies, and avoidance.

Table 4 shows a general decrease in all MCT related factors from session 1 to session 10. In general, the graph shows that treatment was associated with reductions in symptoms, worry, negative- and positive metacognitions, maladaptive coping strategies, and avoidance.

**Table 4:** Changes on GADS-R from session to session.

	Symptoms		Worry		Negative beliefs		Positive beliefs		Coping strategies		Avoidance	
						
	*M*	*SD*	*M*	*SD*	*M*	*SD*	*M*	*SD*	*M*	**SD**	*M*	*SD*
Pre	5.3	1.1	5.5	1.3	5.4	1.7	2.4	2.1	4.3	1.2	3.0	1.3
1	5.4	1.1	5.2	0.9	5.3	1.2	3.0	2.3	4.4	1.0	2.6	1.0
2	4.9	1.3	5.0	1.4	4.4	1.7	2.0	1.7	3.6	1.2	2.0	1.2
3	4.3	1.4	4.2	1.7	3.5	1.6	1.4	1.3	3.2	1.3	1.7	1.1
4	4.1	1.6	3.9	2.0	3.2	1.9	1.2	1.2	2.6	1.6	1.4	1.1
5	3.8	1.8	3.4	1.8	2.1	1.8	0.9	0.9	2.0	1.3	1.1	1.1
6	3.4	1.6	2.5	1.7	1.8	1.8	0.5	0.8	1.6	1.2	0.7	0.6
7	2.4	1.3	1.8	1.2	0.8	1.1	0.5	0.9	1.1	1.0	0.6	0.7
8	2.3	1.8	1.8	1.4	0.6	1.1	0.4	0.7	1.1	1.2	0.6	0.8
9	2.3	2.0	1.8	1.7	0.5	0.8	0.2	0.6	0.8	0.7	0.4	0.5
Post	2.0	1.4	1.3	1.3	0.2	0.3	0.2	0.5	0.7	0.9	0.3	0.6
F-U	1.4	1.2	1.0	1.0	0.1	0.2	0.1	0.1	0.5	0.6	0.2	0.6


*Changes from session to session (pre-treatment to 3-month follow-up) in GAD symptoms, worry, negative- and positive metacognitions, maladaptive coping strategies, and avoidance. All scores are transformed to a 0–8 scale.*

### Comparison With Other GAD Trials

**FIGURE 2:**
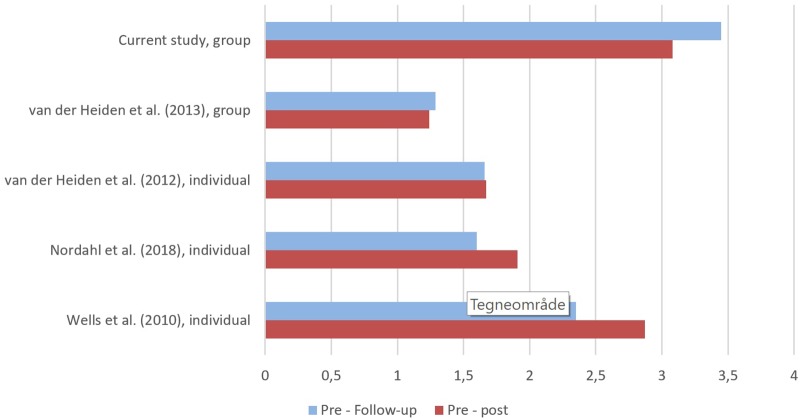
Comparison of uncontrolled effect sizes in generalized anxiety disorder (GAD) trials using metacognitive therapy (MCT). All data are based on intention-to-treat and effect sizes are calculated using pooled standard deviations. All outcomes are assessed using the Penn State Worry Questionnaire (PSWQ).

For benchmarking purposes, uncontrolled effect sizes (all outcome measures using the PSWQ) were compared to the previously mentioned studies of MCT for GAD (Wells et al., 2010; van der Heiden et al., 2012, 2013; Nordahl et al., 2018). Figure 2 shows effect sizes (using pooled standard deviations) from pre-treatment to post-treatment and from pre-treatment to follow-up for the various studies. The results suggested that patients in the current study had obtained large reductions in symptoms of worry that were comparable even with individual MCT for GAD. Patients in the current study had quite high scores on PSWQ at pre-treatment, whereas post-treatment and follow-up scores were comparable with results from individual MCT. *T*-tests comparing the results of the current study with that of Wells et al. (2010) showed that the current study had a significantly higher PSWQ pre-treatment score, *t*(31) = 2.86, *p* = 0.007, while there was no significant difference at post-treatment, *t*(31) = 0.14, *p* = 0.889 and follow-up, *t*(31) = 0.55, *p* = 0.587.

The average number of therapist hours per patient in this study was 6.5 h (10 session × 1.5 h × 2 therapists^∗^5 groups/23 patients = 6.5), which accounts for fewer hours per patient compared to Wells et al. (2010) and van der Heiden et al. (2012) which had 10–12 sessions (45–60 min each) per patient.

## Discussion

The aims of the current study were to evaluate the feasibility and effectiveness of g-MCT for patients with GAD within the context of an ordinary psychiatric clinic. As only a small proportion of patients declined g-MCT in favor of individual MCT and no patients dropped out during treatment, g-MCT appeared to be an acceptable treatment modality. Furthermore, g-MCT was associated with significant reductions in worry and symptoms of anxiety and depression. There were also significant reductions in all MCT related factors such as positive metacognitive beliefs, negative metacognitive beliefs, and maladaptive coping strategies (including avoidance behavior). Session to session ratings indicated that the reduction in symptoms, metacognition, and coping behavior coincided with each other. However, due to the design of the study, the results provide no clarity with respect to causal relationships. In sum, large effect sizes and high recovery rates indicate that g-MCT is an effective treatment for GAD.

With respect to treatment feasibility, 23 patients received treatment, while 22 patients were excluded. GAD not being the primary diagnosis (*n* = 9) was the most common reason for exclusion. Six patients (19 %) declined g-MCT in favor of individual MCT, and three patients (9%) were unable to attend due to scheduling conflicts. Thus, 28% of participants who were offered treatment chose not to participate. This rate is slightly higher compared to a previous RCT study [19.8% (20 of 101 eligible patients)] offering individual treatment (Nordahl et al., 2018). Group treatment could also be less flexible than individual treatment which could exclude patients with set or busy schedules. On the other hand, a positive aspect is that none of the included patients dropped out during treatment, suggesting that g-MCT was accepted by the participants. Furthermore, the average number of therapist hours per patient in this study was 6.5 h, which accounts for fewer hours per patient compared to studies using individual therapy (typically 10–12 sessions). Thus, g-MCT appear to be a cost-effective treatment method.

According to benchmarking analyses, patients in the current study had quite high scores on PSWQ at pre-treatment, while post-treatment and follow-up scores were comparable to previous investigations of individual MCT for GAD (Wells et al., 2010; van der Heiden et al., 2012; Nordahl et al., 2018). The recovery rate (PSWQ) at post-treatment in this study was 65.3%, which is somewhat lower than Wells et al. (2010). This might be explained by the high pre-treatment scores in the current study. However, the recovery rate increased to 78.3% at 3-month follow-up, which is in line with results from individual MCT. The group study of van der Heiden et al. (2013) showed somewhat lower recovery rates than the current study. It could be speculated that this is related to differences in group size (4–6 patients vs. 10–14 patients per group), but it could also be related to therapist factors, as two of their four therapists had not received MCT training. When comparing uncontrolled within effect sizes for studies on MCT for GAD, the current study showed promising results. However, the effect size estimation could be inflated and influenced by the relatively small sample size. The results are also encouraging when compared to recovery rates in CBT. As previously mentioned 50–60% are recovered following CBT for GAD (Fisher and Durham, 1999), and only 38% were recovered in a recent study (Nordahl et al., 2018).

Group-MCT was associated with significant reductions in positive and negative metacognitions. The reduction was greater for the negative metacognitive beliefs than for positive beliefs. A possible explanation could be that patients reported fewer positive than negative metacognitive beliefs at the start of treatment.

Treatment was also associated with reduction in symptoms of depression and comorbidity did not affect treatment outcome. This is an appealing aspect of treatment given the high rate of comorbidity (and overlap in symptoms) between GAD and depression. This finding is also consistent with studies showing that MCT has an effect on comorbid disorders (e.g., Johnson et al., 2017; Capobianco et al., 2018; Papageorgiou et al., 2018). The fact that treatment reduced comorbid symptoms of depression is also consistent with a metacognitive understanding of common underlying psychological processes in emotional disorders, and therefore supports a transdiagnostic utility of MCT.

The study is not without limitations. The most obvious is the open trial design lacking a control group. Therefore, the study is unable to control for random fluctuations, spontaneous recovery, or effect of external variables. Evaluation of treatment effectiveness was also based on self-reported symptoms, which poses certain limitations such as social desirability. However, this effect could also be present for interview based ratings. Diagnostic re-assessment at long term follow-up is ongoing. Another issue is that it was a predominantly a female sample, as well as a probable overrepresentation of patients with comorbid OCD. A strength of the study is however that treatment outcomes were comparable for patients with and without comorbid disorders. Furthermore, there was no official measure of adherence. However, video supervision was conducted with an international expert in MCT and several groups had been conducted for training purposes before the open trial was initiated. Another issue is that diagnostic interviews were not videotaped and there is no measure of inter-rater agreement. Sample size is also an issue for the comorbidity analyses and comparing results across treatment studies is not always straightforward as samples and conditions may vary.

## Conclusion

In conclusion, the results of this study show that g-MCT was a suitable and effective treatment for patients with GAD. Treatment was associated with significant reductions in worry, anxiety, dysfunctional metacognitions, and coping strategies. It was also associated with significant improvement in symptoms of depression, which supports the transdiagnostic effects of MCT. Effect sizes were high and recovery rates were comparable to previous studies. The study supports further evaluation of group-MCT for patients with GAD using larger sample sizes and controlled designs.

## Author Contributions

SH, SS, and PF were responsible for designing the study. SH and GS conducted the therapy. PF supervised the therapists. EB and SS wrote the first draft of the manuscript and conducted statistical analyses. EB and TG were responsible for diagnostic interviews. SS acted as principle investigator and was responsible for getting ethical approval. All authors have contributed in revising the manuscript and approved its submission.

## Conflict of Interest Statement

The authors declare that the research was conducted in the absence of any commercial or financial relationships that could be construed as a potential conflict of interest. The reviewer BF declared a past co-authorship with one of the authors PF to the handling Editor.
